# Pressure-Dependent Meso-Scale Evolution of Dispersed Foam and Field-Application Clogging Mitigation Strategies for EPB Shield Tunnelling Through Highly Cohesive Clay

**DOI:** 10.3390/ma18122716

**Published:** 2025-06-09

**Authors:** Shisen Zhao, Kefeng Peng, Jinliang Bai

**Affiliations:** 1 State Key Laboratory of Intelligent Construction and Healthy Operation and Maintenance of Deep Underground Engineering, China University of Mining and Technology, Xuzhou 210096, China; 2 School of Qilu Transportation, Shandong University, Jinan 250002, China; peng_kefeng@foxmail.com; 3 School of Mechanics and Civil Engineering, China University of Mining and Technology, Xuzhou 210096, China; 02223964@cumt.edu.cn

**Keywords:** clogging mitigation, dispersed foam agent, highly cohesive clay condition, evolutionary mechanisms of bubble size distribution, foam half-life, adhesion characteristic of conditioned clay, optimal foam injection ratio

## Abstract

Clogging in earth pressure balance (EPB) shield tunnelling through highly cohesive strata critically undermines construction efficiency. Conventional foam agents exhibit limited conditioning effectiveness, even with increased dosage. This study developed a dispersed foam agent by combining anionic surfactant (AES) with nonionic dispersant (HDT). The effects of air pressure (0–2 bar) and HDT content (0–10%) on macro-meso characteristics of foam and adhesion characteristics of conditioned soil were quantified through an evolutionary mechanism investigation of the bubble size distribution of foam, half-life measurements, and mechanical tests on conditioned soils. Results demonstrated that the influence of HDT content on foam exhibited pressure-dependent behavior. Under 0 bar within 0–10 min, HDT increased the proportion of small bubbles while marginally reducing the mean radii. Although HDT accelerated the degradation of small bubbles, it extended the foam half-life. Conversely, under 1 or 2 bar, HDT demonstrated opposite effects on these parameters. The cohesion of conditioned clays was reduced to 1.8–4.3 kPa, and adhesion amounts decreased to 10–15 g, significantly mitigating clogging risks. The optimal injection ratio of dispersed foam was determined for different pressures and clays. Engineering application in an EPB shield tunnelling section of Jinan Metro successfully resolved clogging issues, demonstrating the effectiveness of dispersed foam agent.

## 1. Introduction

With the implementation of the national “the Belt and Road” strategy and the “14th Five-Year Plan” comprehensive transportation development plan, the transportation infrastructure in China has been developing rapidly [1]. Many tunnels are currently under construction. Owing to these advantages, earth pressure balance (EPB) shield tunnelling technology is widely used in fields such as highways, railways, municipal roads, urban rail transit, and hydraulic tunnels. More than 90% of shield tunnels passing through soft-soil areas worldwide use EPB shield tunnelling. When EPB shield tunnelling passes through cohesive strata, blockage is prone to occur. Adhesion between clay minerals and mechanical surfaces is a key factor in clogging [2]. The occurrence of clogging can be significantly inhibited by reducing the adhesion between the soil and the mechanical surface [3,4,5,6]. Therefore, using muck soil improvement technology to adjust the mechanical properties of muck soil appropriately is a necessary technical measure to ensure efficient shield tunnelling. Therefore, the use of conditioners to improve the mechanical properties of excavated soil is necessary to ensure efficient shield tunnelling.

Scholars have conducted extensive research on soil conditions in clay strata. Yuanli Wu et al. proposed a method that employs microscopic observation of the mesoscopic characteristics of foam to evaluate its macroscopic properties, analyzing the soil improvement effects of foam generated by foaming agents from the mesoscopic level [7,8]. Xinyu Ye et al. [9] and Daniele Peila et al. [10] conducted slump tests to explore the fluidity of soil conditioned by foam. R. Zumsteg et al. [11,12] analyzed the characteristics of the shear resistance of foam-conditioned soil by conducting tests of empirical stickiness ratio and pressurized vane shear. Daniela G.G. de Oliveira et al. [13] evaluated the fluidity and clogging characteristics of conditioned soil using an improved empirical stickiness ratio test. Daniele Peila et al. [14] analyzed the influence of foam agent content and soil moisture content on soil condition by the self-designed dynamic adhesion test system. Daniele Martinelli et al. [15] studied the behavior of conditioned soil under pressure conditions with triaxial tests and evaluated the conditioned effect in combination with slump tests. Bin Zhu et al. [16] investigated the transport characteristics of soil in the screw conveyor model before and after the condition and optimized the reasonable value range of slump. Zhongtian Chen et al. [17] described the development process of the clogging phenomenon with a small shield and analyzed the relationship between tunnelling parameter changes and clogging. Yong Fang et al. [18] and Bin Zhuo et al. [19] studied adhesion stress before and after soil conditioning and the contact angles between clay minerals and surfactant solution at the microscopic scale, and the conditioned mechanism of foam on clay was revealed.

Most studies have focused on the effects of foam agents. However, actual engineering shows that the effect of foam agents is poor when the content of adhesive particles in the soil is high. Even if the foam dosage is increased, the effect is limited, and it causes other problems (such as increasing the muck discharge volume). Reducing adhesion conditioners suitable for highly sticky soils have been developed, and verifying their effectiveness has become a new research focus. Pengfei Li et al. [20] and Shuying Wang et al. [21] studied the effect of dispersants on the Atterberg limit of clay and revealed the influence of dispersants on the adhesion of clay. Yong Fang et al. [22] investigated the influence of the foam agent and dispersant on the Atterberg limit, adhesion, and tangential adhesion of the conditioned soil by improved pull-out tests. Zeen Wan et al. [23] analyzed the condition effect of anti-clay agent and foam agent on the dynamic cohesion of weathered mudstone by the dynamic cohesion test. Bin Zhuo et al. [24] studied the soil fluidity conditioned by foam agent and dispersant with the slump test and rotating torque test.

Despite their potential applications, systematic investigations into foam agents with dispersive constituents have been limited in the existing literature. In response to the research background outlined above, this study aims to develop a dispersed foam agent and formulate clogging mitigation strategies for clays with different plasticity indices (PIs), thereby reducing clogging risks during EPB shield tunnelling through highly cohesive clay strata. To achieve this goal, the following four sequential intermediate goals must be met: ① Conduct mesoscopic characterization of foam evolution mechanisms and stability characteristics through time-resolved bubble size analysis under controlled HDT concentrations (0–10%) and ambient air pressures (0–2 bar), utilizing microscopy and digital image processing. ② Optimize the formulation of the dispersed foam agent by coupling an anionic surfactant (AES) with a nonionic dispersant (HDT), comprehensively considering foam evolution mechanisms and stability characteristics. ③ Quantify adhesion reduction using custom-designed unsaturated soil direct shear tests and rotary cutterhead simulation tests, evaluating the adhesion characteristics of conditioned clays across plasticity indices (PI = 20–50). ④ Establish injection guidelines based on the dispersed foam agent for clays of varying plasticity indices and validate their effectiveness through field application in a shield tunnelling section of Jinan Metro Line 6.

## 2. Materials and Methods

The soil conditioning mechanism of foaming agents primarily involves two aspects: (1) the generated foam is injected into the soil to enhance the compressibility and flowability of the conditioned soil, thereby reducing its shear strength; (2) surfactant molecules in the foam agent adsorb onto the surface of soil particles, forming a lubricating liquid film that reduces soil adhesiveness. However, when applied to highly cohesive soils, conventional foam agents have limited effectiveness in reducing soil adhesion, making it difficult to prevent clogging issues.

To address this limitation, a nonionic dispersant is introduced. Once injected into the soil along with the foam, dispersant molecules adsorb onto the surface of clay particles and contribute in two ways: (1) by ionizing functional groups, they increase the negative surface charge density of clay particles, thereby weakening van der Waals forces between them; and (2) by adsorbing at different sites on the clay surface compared to surfactants, the two types of condition agent molecules act synergistically to form a more extensive lubricating film. This significantly reduces interparticle adhesion in clay and effectively mitigates clogging problems.

Building on this conceptual framework, this study developed a dispersed foam agent suitable for highly cohesive strata by using an anionic surfactant (sodium fatty alcohol ether sulfate; AES) as the primary foaming agent and incorporating a nonionic dispersant (HDT) as a performance enhancer. The agent was intended for soil conditioning during EPB shield tunnelling through highly cohesive soils.

### 2.1. Experimental Materials

#### 2.1.1. Surfactant and Dispersant

The surfactant used in this study was an anionic surfactant, sodium fatty alcohol ether sulfate (AES), and the performance enhancer was a nonionic dispersant, HDT. Both were supplied by Shandong Hongyu Engineering Technology Co., Ltd. (Dezhou, China). In the preparation of the foam agent, the AES content was fixed at 15%, while the HDT content was varied at 0.0%, 2.0%, 4.0%, 6.0%, 8.0%, and 10.0%. Additionally, 0.1% of a foam stabilizer was added. The three components were sequentially dissolved in water to produce a homogeneous and stable foam agent solution, which was then left to stand for 24 h before use. The necessary properties of the AES, HDT, and foam stabilizer are shown in Table 1.

#### 2.1.2. Clay Specimen

Highly cohesive clay refers to soil with a plasticity index (PI) exceeding 20 [25]. Synthetic, highly cohesive clay specimens were prepared for this study. Bentonite, illite powder, and kaolin were blended in controlled mass ratios to prepare highly cohesive clays with target PI values of approximately 20, 30, 40, and 50, respectively. The specific proportions and water contents are detailed in Table 2. The synthesis procedure involved the following steps: ① Homogenization of dry powders in a planetary mixer (30 min at 60 rpm); ② Gradual addition of water during mixing; ③ Sealing the mixture at 25 °C for 24 h to ensure moisture equilibrium.

All three clay minerals were procured from Shandong Hongyu Engineering Technology Co., Ltd. (Dezhou, China). The key properties are summarized in Table 3. The particle size distribution (PSD) was determined using a BT-9300SE instrument supplied by Dandong Baite Instrument Co., Ltd. (Dandong, China). The PSDs of the four clay samples are provided in Table 4.

### 2.2. Test Condition

The pressure in the working chamber typically does not exceed 2 bar during EPB shield tunnelling. Therefore, the test air pressures were set at 0 bar, 1 bar, and 2 bar, referring to gauge pressure (hereafter referred to simply as “pressure”, *p*); under this definition, atmospheric pressure corresponds to 0 bar. The test temperature was maintained at 25 °C.

The AES content (*c_a_*) and HDT content (*c_d_*) refer to the mass proportions of AES and HDT in the dispersed foam agent, respectively. The foam solution refers to the diluted solution formed by mixing the dispersed foam agent with water at a specified concentration, which is then used to generate foam. In EPB shield tunnelling, the foam agent concentration typically ranges from 2% to 10% (i.e., the mass proportion of foam agent in the foam solution), and the foam expansion ratio (FER) is generally set between 5 and 30. Accordingly, in this study, the foam agent concentration was set at 3%, and the FER was set at 15.

In the adhesion test of conditioned soil, the foam injection ratio (the volume ratio of injected foam to soil; FIR) was varied as follows: 0%, 10%, 20%, 30%, 40%, 50%, 60%, 70%, 80%, 90%, and 100%.

### 2.3. Test Apparatus and Procedure

#### 2.3.1. Foam Evolution Mechanism

**Figure 1:**
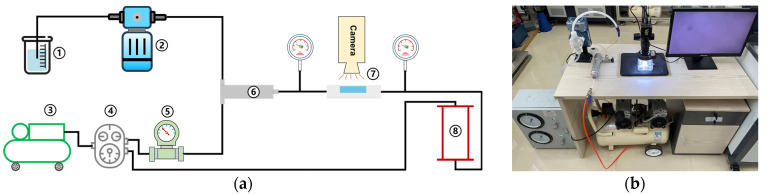
Schematic diagram of foam test system: (**a**) system design drawing; (**b**) picture of the system.

**Figure 2:**
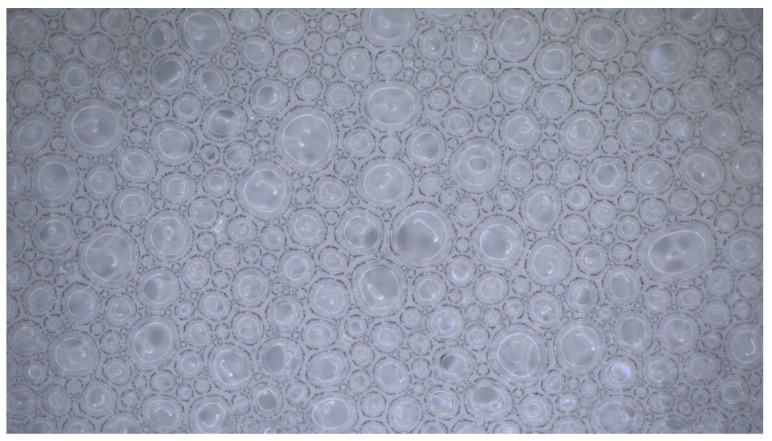
The foam image (for illustration only).

A self-developed foam testing system was used to measure the time-dependent variation of foam bubble size distribution and foam half-life. As shown in Figure 1, this system performs three main functions: foam generation, bubble size analysis, and foam half-life measurement [7]. It consists of a foaming solution pump ②, air supply devices ③, a pressure control device ④, a gas flow meter ⑤, a foam generator ⑥, an observation window with a microscope ⑦ (see Figure 1), and a foam degradation column ⑧. The foam generator is filled with 2 mm glass beads. The microscope used in the foam test system is the Bosheng BC1201E measuring microscope, which has the functions of magnification (20–200 times) and photographing with a high resolution (2 μm/pixel). The foam image taken is shown in Figure 2. The inner diameter of the foam degradation column is 100 mm, and the height is 800 mm. It is composed of acrylic material.

This integrated apparatus enables (a) mesoscale analysis of foam evolution mechanisms (e.g., bubble size distribution dynamics and half-life decay) via real-time microscopy and digital image processing (ImageJ 1.54g), surpassing macroscopic approaches (e.g., expansion ratio) in precision; and (b) pressure-controlled foam characterization under varied ambient pressures (0–2 bar), addressing the gap in existing methods limited to atmospheric conditions. The experimental procedure can be referenced in Appendix A.1.

#### 2.3.2. Adhesion Characteristics Test of Conditioned Soil

**Figure 3:**
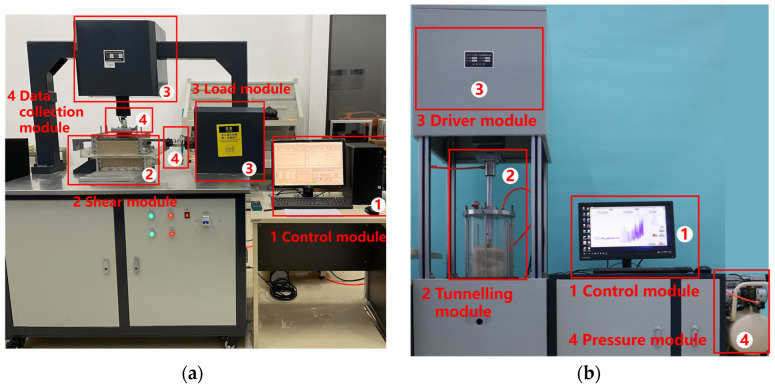
Adhesion characteristics test apparatus: (**a**) unsaturated soil direct shear system; (**b**) cutterhead rotation adhesion testing system.

The adhesion characteristics test of conditioned soil included cohesion testing and cutterhead rotation adhesion testing. The cohesion testing is conducted using an unsaturated soil direct shear system (see Figure 3a), which consists of a control module, shear module, load module, and data collection module, and is used to determine the cohesion of the clay specimen [26]. The cutterhead rotation adhesion testing is conducted using a self-developed cutterhead rotation adhesion testing system (see Figure 3b), which consists of a control module, tunnelling module (soil chamber, mini cutterhead), driver module, and pressure module [27]. This apparatus can replicate the working chamber pressure (0–2 bar) of an EPB shield, enabling the quantification of adhesion characteristics of conditioned clay under excavation conditions. This capability thereby provides direct insight into the effectiveness of clogging mitigation measures. The experimental procedure can be referenced in Appendix A.2 and Appendix A.3.

## 3. Results and Discussion

### 3.1. Effect of HDT Content on Foam Evolution Mechanism Under Different Pressures

#### 3.1.1. Bubble Size Distribution and Temporal Evolution of Foam

**Figure 4:**
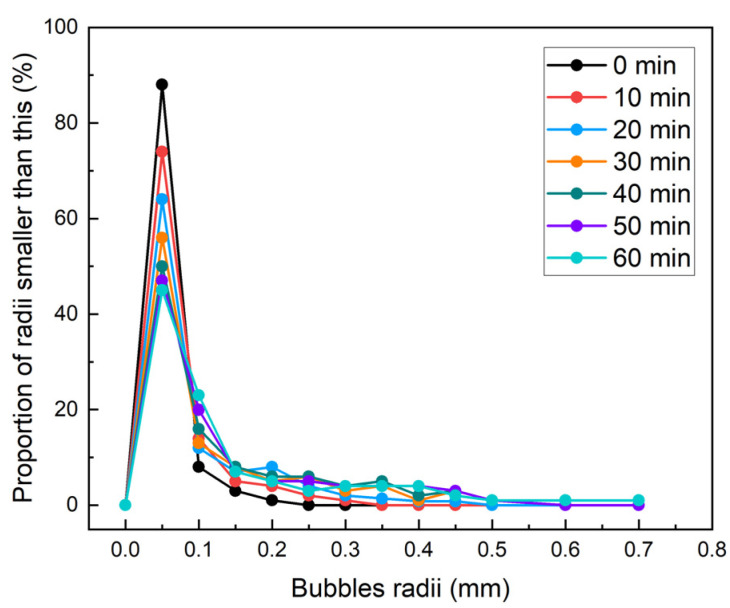
The evolution of bubble size distribution with *p* = 2 bar and *c_d_* = 4.0%.

**Figure 5:**
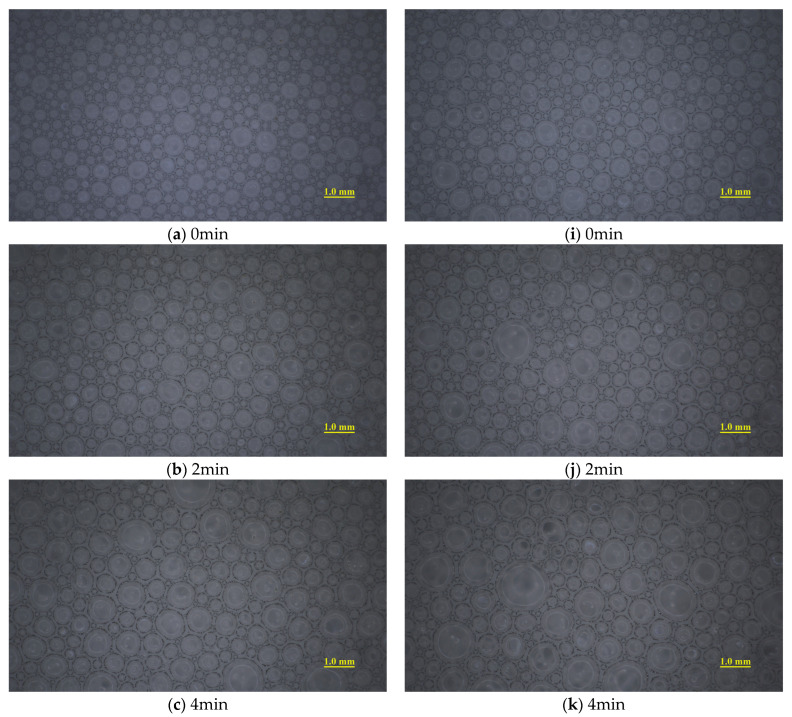

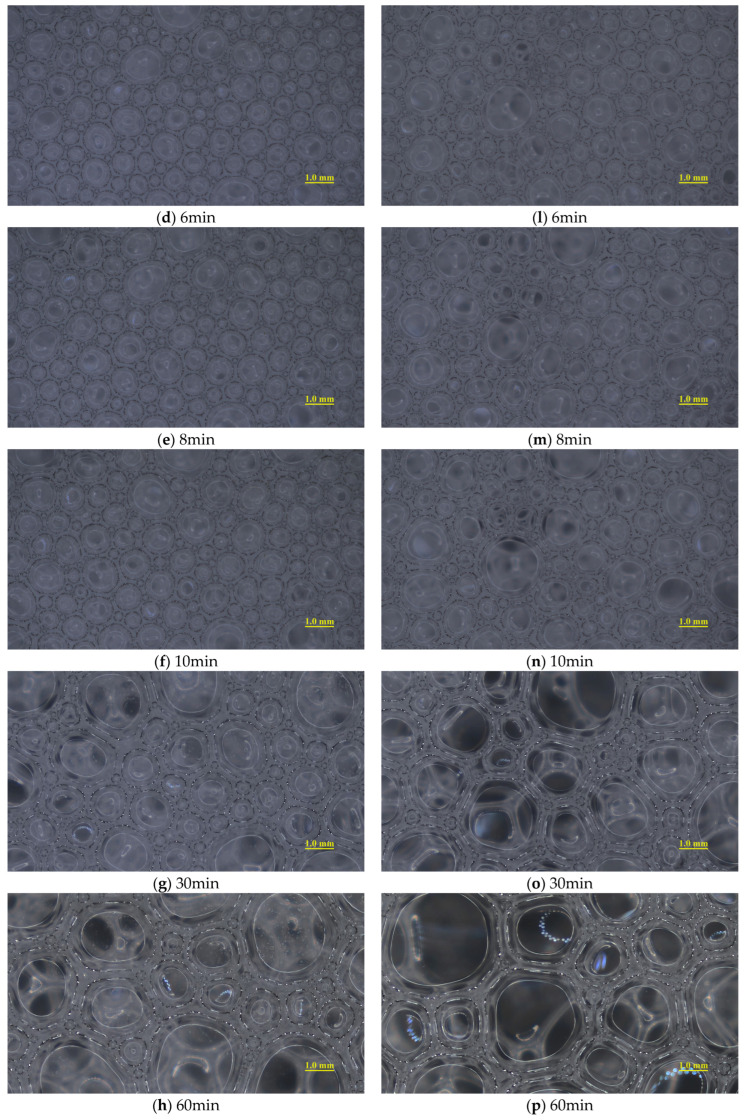
Evolution images of bubble size distribution with *p* = 1 bar: (**a**–**h**): *c_d_* = 0.0%; (**i**–**p**): *c_d_* = 4.0%.

Figure 4 shows the evolution of bubble size distribution with *p* = 2 bar with *c_d_* = 4%. As time progresses, the distribution curve gradually flattened and shifted to the right, indicating that the overall bubble size of the foam increases over time. The bubble size distributions under different pressures and *c_d_* exhibited similar patterns (Figure 5). During the experimental period (1 h), bubbles with a radius of less than 0.05 mm (referred to as “small bubbles”) accounted for a very high proportion. Wu et al. [7] suggested that small bubbles significantly slow down the foam drainage rate, playing a key role in soil conditioning. Therefore, the evolution mechanism of foam in this bubble size range was analyzed in detail.

**Figure 6:**
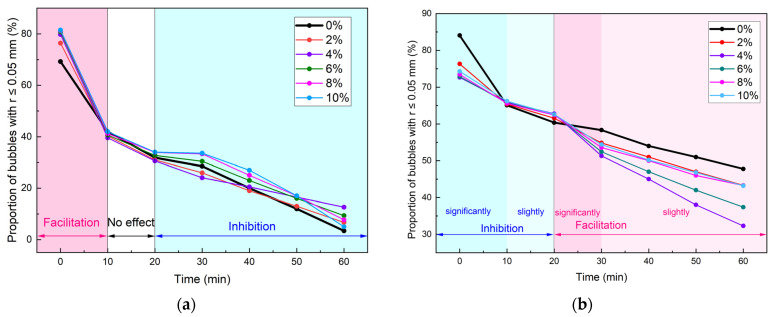
Effect of *c_d_* on the evolution mechanism of small bubbles under different *p*: (**a**) 0 bar; (**b**) 1 bar.

Figure 6 compares the influence of *c_d_* on the evolution mechanism of small bubbles under 0 bar and 1 bar pressure conditions. When *p* = 0 bar, HDT accelerated the degradation of small bubbles during the initial 10 min. This acceleration effect intensified progressively, with *c_d_* increasing from 0.0% to 4.0%, but remained nearly constant at higher *c_d_*. No significant HDT influence was observed during 10–20 min, while bubble degradation inhibition emerged after 20 min.

Conversely, at 1 bar pressure, HDT markedly inhibited foam degradation within the first 10 min, with stabilization of inhibition effects above *c_d_* = 4.0%. Mild inhibition persisted during 10–20 min, followed by pronounced degradation acceleration from 20–30 min. During 30–60 min, the acceleration effect diminished to marginal levels. Similar trends were observed at 2 bar pressure.

**Figure 7:**
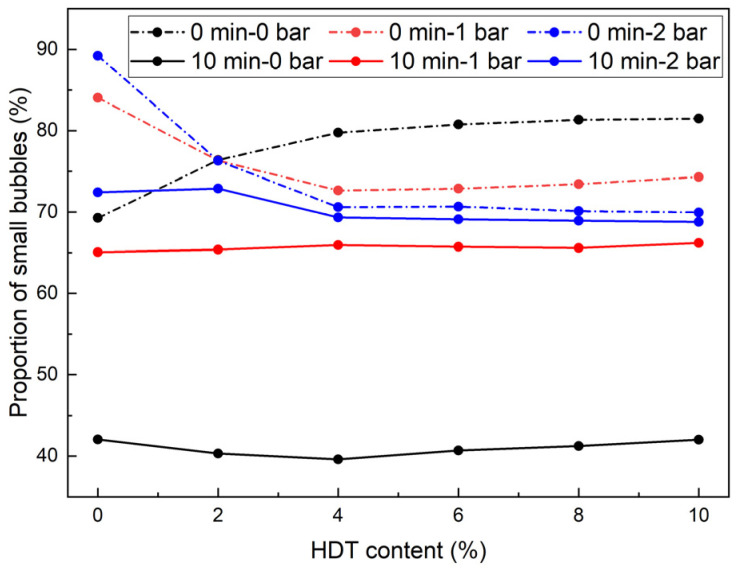
Influence of different *c_d_* on the proportion of small bubbles in 0–10 min.

In the conditioning process of clay, the first 10 min after foam injection into the clay are crucial for changes in clay mechanical properties. Figure 7 shows the impact of different *c_d_* under various p on the proportion of small bubbles during 0–10 min. It could be observed that the *p* determined the effect of HDT on the small bubble proportion, which was completely opposite under different *p*. Under 0 bar conditions: At 0 min, HDT presence increased the small bubble proportion from 69.27% (*c_d_* = 0%) to approximately 80% (*c_d_* = 4%) with stabilization. After 10 min of stabilization, this proportion exhibited an initial slight decrease, followed by stabilization across varying *c_d_* levels. Under 1 bar conditions: Contrary to 0 bar behavior, the small bubble proportion decreased from 84.05% (*c_d_* = 0%) to ~74% (*c_d_* = 4%) at 0 min before stabilizing. After 10 min of evolution, HDT exerted completely reversed effects compared to initial-phase observations, with similar patterns observed at 2 bar. The observation of dominant small bubbles (74–89% under 1–2 bar) aligns with [7,28], which emphasized that small bubbles critically decelerate foam drainage. However, the pressure-dependent inversion effect of HDT (increasing small bubbles at 0 bar vs. decreasing at 1–2 bar) represents a novel finding not reported elsewhere.

The above analysis revealed that at 0 bar, the introduction of HDT increased the initial small bubble population (0 min) while simultaneously accelerating their degradation rate. Nevertheless, throughout the experimental period, the HDT-containing conditioners maintained significantly higher small bubble quantities compared to AES-only conditioners, with 4% identified as the optimal HDT content.

Under pressurized conditions (1 or 2 bar), although HDT reduced the initial small bubble population (0 min), it notably inhibited bubble degradation during 0–10 min. Specifically, at 10 min, HDT-containing conditioners exhibited higher small bubble proportions than AES-only conditioners. The critical role of HDT lies in bubble stabilization, with optimal concentrations being 2% or 4% under these pressure conditions. Consequently, through a comprehensive evaluation of HDT’s effects on bubble size distribution and temporal evolution patterns, the optimal *c_d_* is determined to be 4%.

#### 3.1.2. Mean Radii Evolution of the Foam

**Figure 8:**
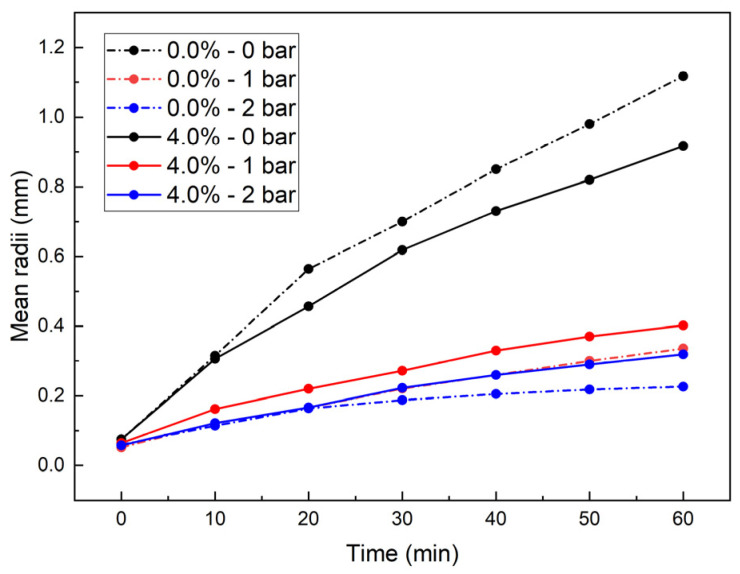
Time-varying regularity of mean radii.

The average bubble radius significantly affects the toughness of the foam: smaller mean radii indicate better foam toughness, which reduces the likelihood of rupture when encountering highly absorbent clay and enhances the effectiveness of soil conditioning. Figure 8 illustrates the evolution of the mean radii under different *p* when *c_d_* = 0.0% and *c_d_* = 4.0%. Over time, the mean radii increased, following a power-law trend, which was consistent with existing research findings [7]. The mean radii decreased with increasing *p*, and foams containing HDT still followed the traditional foam degradation mechanism [29]. Under 0 bar, the addition of HDT reduced the mean radii, whereas under 1 bar or 2 bar, HDT addition increased the mean radii.

**Figure 9:**
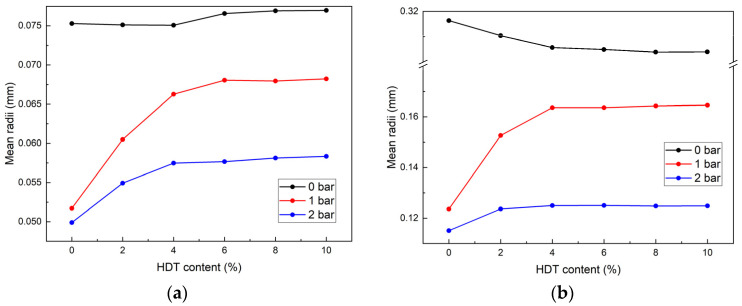
The influence of *c_d_* on the mean radii under different time and *p*: (**a**) 0 min; (**b**) 10 min.

The adhesion reduction process of soil condition in EPB shield tunneling primarily occurs during the initial condition stage. This study, therefore, focused on analyzing the characteristic variations in bubble mean radii during the first 10 min. Figure 9 illustrates the influence of *c_d_* on bubble mean radii under different *p* during this critical period.

At 0 min: Under 0 bar conditions, the mean radii demonstrated an initial slight decrease followed by a gradual increase and subsequent stabilization with increasing *c_d_*. The minimum mean radii were observed at *c_d_* = 4%. At 1 bar and 2 bar conditions, the mean radii exhibited a positive correlation with *c_d_* within the 0–4% concentration range, followed by stabilization at higher *c_d_* levels.

At 10 min: The 1 bar and 2 bar conditions maintained identical variation patterns to initial observations (t = 0 min). Under 0 bar conditions, the mean radii displayed a distinct response to *c_d_*, showing an initial reduction followed by stabilization with increasing *c_d_*.

The power-law growth trend of mean radii (Figure 8) is consistent with Stevenson’s foam degradation theory [29]. However, the HDT-induced reduction in mean radii at 0 bar contrasts with conventional surfactants, demonstrating the unique role of dispersants.

Based on the above analysis, the following conclusions can be drawn. At 0 bar pressure, HDT introduction effectively reduces bubble mean radii, achieving the minimum mean radii at *c_d_* = 4% throughout the 0–10 min monitoring period, with stabilization observed beyond this concentration threshold. At 1 bar or 2 bar, the introduction of HDT resulted in an increase in the bubble mean radii. Consequently, the optimal *c_d_* for these pressure regimes was identified as 2% or 4%, balancing the required bubble mean radii and the introduction of HDT.

### 3.2. Effect of HDT Content on Foam Half-Life Under Different Air Pressures

**Figure 10:**
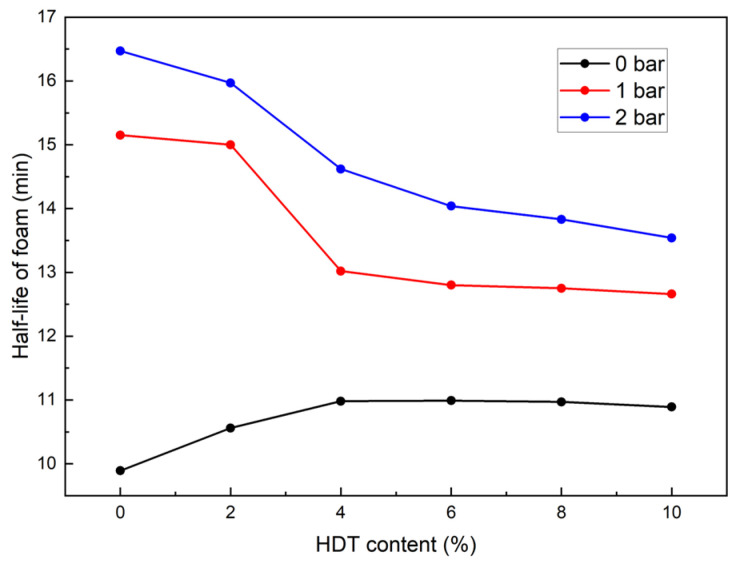
Variation of half-life of foam under different *c_d_* and *p*.

The effect of *c_d_* on foam half-life under different *p* is shown in Figure 10. The influence of *c_d_* on foam half-life is dependent on the *p*. Under 0 bar pressure, as *c_d_* increased from 0% to 4%, the half-life gradually increased from 9 min 50 s to 11 min. However, with further increases in *c_d_*, the half-life slightly decreased to approximately 10 min 50 s. Under 1 bar or 2 bar pressure, the half-life decreased progressively with increasing *c_d_*; for example, from 15 min 9 s to 12 min 39 s at 1 bar, and from 16 min 29 s to 13 min 30 s at 2 bar. Previous studies have shown that a half-life of more than 7 min is sufficient to meet the requirements of soil conditioning in EPB shield tunnelling [12]. The foam half-life (9.5–16.5 min) exceeds the threshold for effective EPB conditioning established by [30,31,32]. The pressure-governed HDT effects (extending half-life at 0 bar but shortening it at 1–2 bar) are unprecedented in existing studies [10,33]. All tested *c_d_* values in this study satisfied this requirement. 

The variation in foam half-life can be analyzed in conjunction with the foam microstructural evolution mechanisms discussed above. Under 0 bar pressure, the addition of HDT increases the proportion of small bubbles from 69.27% to 81.5%, while the mean radii are close to 76 μm. Given a constant foam volume, this indicates a significant increase in the total number of bubbles. Since the foam half-life in this study is closely related to gravitational drainage, the increase in small bubbles notably extends the drainage path length under gravity, thereby prolonging the drainage time—that is, extending the half-life of foam.

At 1 bar or 2 bar pressure, the addition of HDT leads to a gradual decrease in the proportion of small bubbles: from 84.05% to 74.03% at 1 bar, and from 89.22% to 76.31% at 2 bar. Meanwhile, the mean radii increase from 51.71 μm to 68.24 μm (1 bar) and from 49.90 μm to 58.34 μm (2 bar). The reduction in the proportion of small bubbles, along with the increase in mean radii, shortens the drainage path under gravity, thereby resulting in a shorter foam half-life.

During the soil conditioning process in EPB shield tunneling, our experimental results demonstrate that the proportion of small bubbles in foam exerts the most significant influence on condition effectiveness, followed by foam half-life, while the mean radii show a relatively minor impact. Comprehensive analysis reveals that when *c_d_* is maintained at 4%, the synergistic interaction between HDT and AES produces foam with optimal performance across various air pressure conditions. Consequently, the formulated dispersed foam agent was developed with the following composition: AES (15%), HDT (4%), foam stabilizer (0.1%), and water (80.9%).

### 3.3. Effects of Dispersed Foam Agents on the Adhesion Properties of Conditioned Soil

#### 3.3.1. Effects of Dispersed Foam Agents on Soil Cohesion

**Figure 11:**
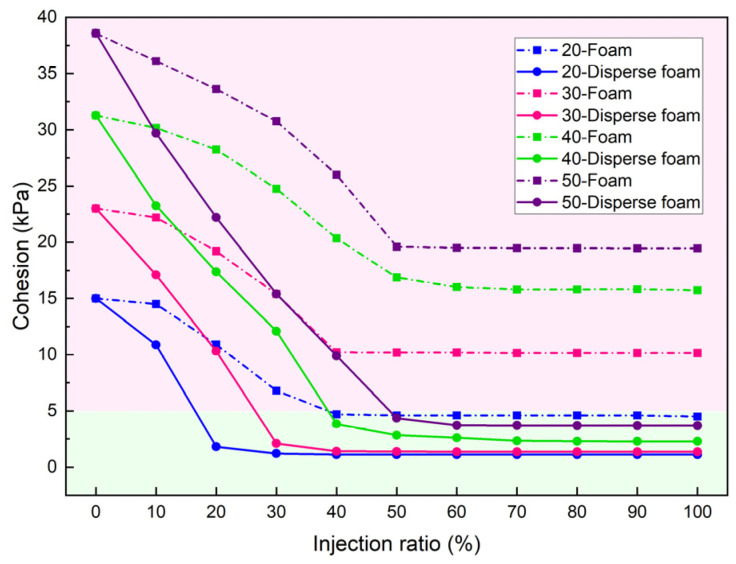
Variation of cohesion under different FIRs.

Figure 11 presents the cohesion variation curves of clays with different PIs after conditioning under 0 bar pressure. In this study, the developed foam agent was designated as the dispersed foam agent, while the surfactant-only formulation was referred to as the foam agent. As shown in Figure 11, the cohesion of conditioned soils demonstrated a characteristic pattern of initial reduction followed by stabilization with increasing FIR. Notably, clays with higher PIs required greater FIR values to achieve minimum cohesion levels.

For foam agents, the cohesion variation of clay with different PIs was as follows: ① for soil with PI = 20, cohesion decreased from 15.12 kPa to 4.7 kPa (FIR from 0% to 40%), then stabilized; ② for PI = 30, cohesion dropped from 23.04 kPa to 10.2 kPa (FIR from 0% to 40%), then stabilized; ③ for PI = 40, cohesion dropped from 31.29 kPa to 16.0 kPa (FIR from 0% to 50%), then stabilized; and ④ for PI = 50, cohesion decreased from 38.59 kPa to 19.5 kPa (FIR from 0% to 50%), then stabilized. The experimental results demonstrated that foam-conditioned clays with a PI other than 20 exhibited cohesion values exceeding 5 kPa, indicating significantly higher clogging potential. 

In contrast, soils conditioned with dispersed foam agents showed significantly lower cohesion: ① for PI = 20, cohesion decreased from 15.12 kPa to 1.8 kPa (FIR from 0% to 20%), then stabilized; ② for PI = 30, cohesion decreased from 23.04 kPa to 2.1 kPa (FIR from 0% to 30%), then stabilized; ③ for PI = 40, cohesion dropped from 31.29 kPa to 2.8 kPa (FIR from 0% to 50%), then stabilized; and ④ for PI = 50, cohesion decreased from 38.59 kPa to 4.3 kPa (FIR from 0% to 50%), then stabilized. At these levels of cohesion, the probability of clogging was extremely low. The dispersed foam agent demonstrated excellent effectiveness in preventing clogging.

#### 3.3.2. Effect of Dispersed Foam Agents on the Adhesion Amounts of Rotating Cutterheads

**Figure 12:**
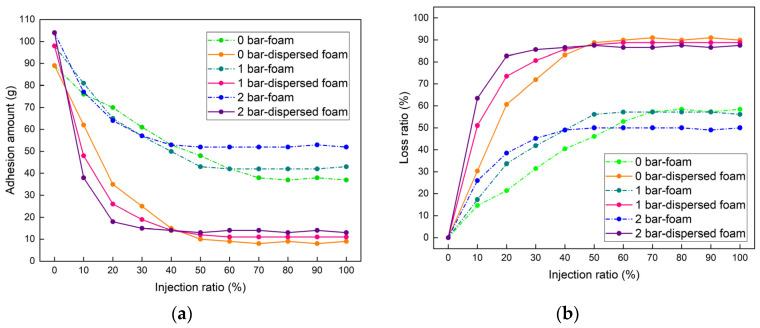
Adhesion on the cutterhead: (**a**) adhesion amount; (**b**) loss ratio of adhesion amount.

Figure 12 shows the experimental results for soil with PI = 40. It could be observed that the adhesion decreases with the increase in FIR under different *p*. For the foam-conditioned soil, when the *p* was 0 bar, as the FIR increased to 70%, the adhesion amount decreased from 89 g to 38 g, a reduction of 57.30%, and then remained almost constant with further increases in the FIR. When the *p* was 1 bar, as the FIR increased to 50%, the adhesion amount decreased from 98g to 43 g, a reduction of 56.12%, and then remained almost constant with further increases in the FIR. When the *p* was 2 bar, as the FIR increased to 40%, the adhesion amount decreased from 104g to 53g, a reduction of 49.04%, and then remained almost constant with further increases in the FIR. As the *p* increased, the optimal FIR for foam-conditioned soil continued to decrease. As shown in Figure 12b, before reaching the optimal FIR for 2 bar pressure, the loss ratio of adhesion amount for the same FIR was as follows: 2 bar > 1 bar > 0 bar.

**Figure 13:**
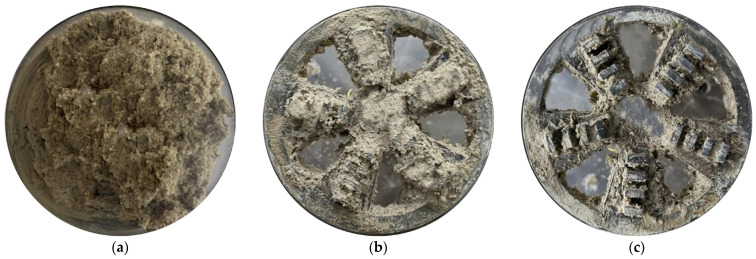
The results of adhesion amount with *p* = 1 bar: (**a**) unconditioned clay; (**b**) clay conditioned by foam agent (FIR = 80%); (**c**) clay conditioned by dispersed foam agent (FIR = 40%).

HDT significantly reduced the adhesion amount on the cutterhead. When the *p* was 0 bar, as the FIR gradually increased to 50%, the adhesion amount decreased from 89 g to 10 g, with a reduction rate of 88.76%, and then remained almost unchanged as the FIR increased. When the *p* was 1 bar, as the FIR gradually increased to 40%, the adhesion amount decreased from 98 g to 14 g, with a reduction rate of 85.71%, and then remained almost unchanged as the FIR increased. When the *p* was 2 bar, as the FIR gradually increased to 30%, the adhesion amount decreased from 104 g to 15 g, with a reduction rate of 85.58%, and then remained almost unchanged as the FIR increased. Compared to the condition method of conventional foam agents, the dispersed foam agent significantly reduced the adhesion amount on the cutterhead. The reduction rate of adhesive clay was not less than 85% for all conditions. As shown in Figure 13, clay conditioned by the foam agent still formed clay accumulation zones on the cutterhead, further leading to clogging. However, the dispersed foam agent significantly reduced the adhesion amount of clay, preventing the clogging. Further detailed tests revealed that for the PI = 40 clay sample, the optimal FIRs under different *p* were 53% (0 bar), 44% (1 bar), and 32% (2 bar).

**Figure 14:**
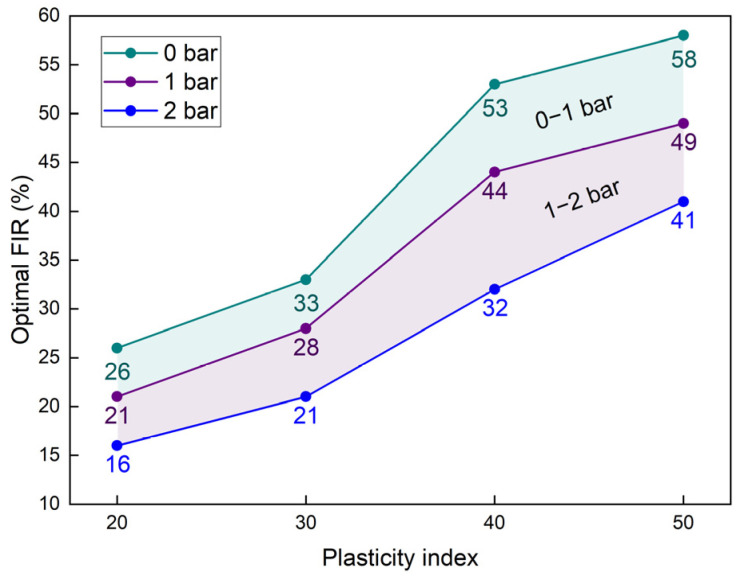
The optimal FIR.

Figure 14 presents the optimal FIRs for clays with different PIs. The experimental data reveal a positive correlation between PI values and their corresponding optimal FIR. Under these optimized injection conditions, the adhesive soil accumulation on cutterheads was effectively controlled below 15% in all test cases. For engineering scenarios where either air pressure conditions or PI values fall between the plotted data points, the established interpolation method provides a reliable optimal FIR determination. This systematic diagram serves as a practical implementation framework, providing operational guidance for the field application of the dispersed foam agent in EPB shield tunnelling projects.

## 4. Engineering Application

### 4.1. Project Overview

**Figure 15:**
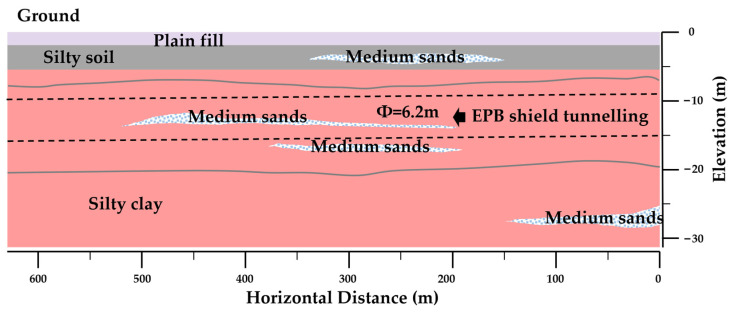
Geological profile of the section; the horizontal scale is 1:4000 and the vertical scale is 1:600.

The Jinan Metro Line 6 project uses a 6.2 m diameter EPB shield for construction in a certain shield tunnel section. The tunnel passes through a silty clay layer (as shown in Figure 15, including 62% silt (0.002–0.075 mm), 35% clay (<0.002 mm), and 3% fine sand), with a PI of 26.5 and a natural water content of 40%.

### 4.2. Application of Dispersed Foam Agent

The tunnel initially used a traditional foam agent with a concentration of 5% for soil conditioning, with an FIR of 50%. When the excavation reached the 48th ring, the torque of the cutterhead quickly increased to 3000 kN·m, and the thrust increased to 12,000 kN, with significant fluctuations. This was considered clogging. After cleaning, the original soil conditioning scheme continued to be used until the 150th ring, during which the cutterhead torque and thrust remained high with significant fluctuations.

The dispersed foam agent was then selected for conditioning, with a concentration of 3% and an FER of 15. Given that the PI of the soil was 26.5 and the soil chamber working pressure was 1.5 bar, and considering the lower water content of the in situ soil compared to the experimental samples, the foam injection ratio was set to 30%.

**Figure 16:**
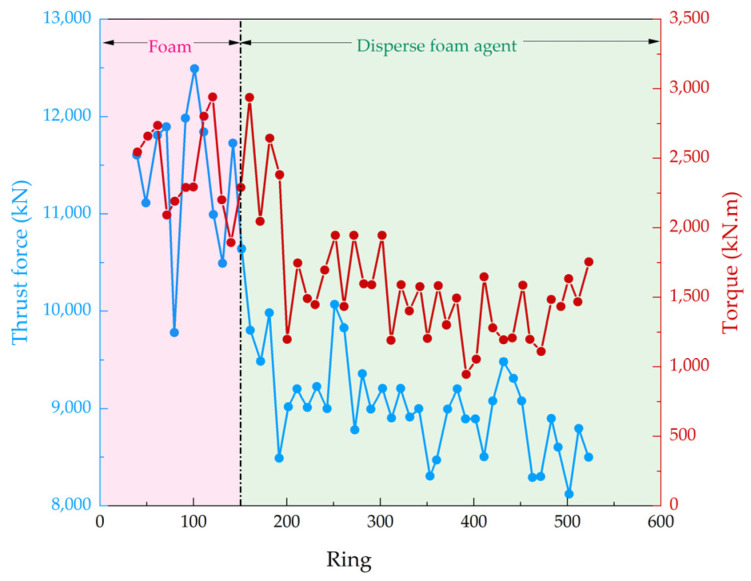
Thrust and torque of shield tunneling.

After switching to the new conditioning scheme, the cutterhead torque rapidly decreased to around 1750 kN·m, and the thrust dropped to around 9000 kN, with smaller fluctuations (see Figure 16). The tunnelling efficiency increased from an average of 3 rings/day to 7 rings/day, significantly improving the excavation efficiency. No clogging occurred until the tunnel was completed. 

Based on the above engineering application, it can be observed that the dispersed foam agent developed in this study eliminated shield clogging issues at an FIR of 30%. This performance is superior to the conditioning approach using a foam agent (FIR = 70%) combined with an anti-clay agent reported by Wan et al. [23] and also outperforms the FIR range of 40–50% required by Lu et al. [34] (as shown in Table 5). Furthermore, the tunnelling speed of the shield machine in the project underpinning this study increased by 133%. This improvement significantly exceeds the tunnelling speed of 3.6 mm/min (approximately 1.5 rings/day) reported by Lu et al. [34] and the 6.5% tunnelling speed increase achieved by Jin et al. [35]. The dispersed foam agent demonstrated excellent clogging prevention effects, and its effectiveness was validated.

## 5. Conclusions

This study investigates the effects of nonionic dispersant (HDT) on foam characteristics (bubble size distribution and half-life) of an anionic surfactant (AES) under varying pressure conditions. A dispersed foam agent was developed and evaluated through cohesion measurements and cutterhead adhesion tests on conditioned clays. Operational guidelines of the dispersed foam agent were established based on PI and working pressures. Field validation at Jinan Metro Line 6 demonstrated effective clogging mitigation. Key conclusions are as follows:

(1) Influence of HDT on small bubble evolution exhibited pressure-dependent characteristics, primarily within the initial 10 min. At 0 bar: HDT increased small bubble proportion (stabilizing with concentration) while accelerating early-stage degradation (0–10 min). At 1 bar or 2 bar: HDT decreased small bubble proportion (stabilizing with concentration) while inhibiting early degradation. Subsequent small bubble evolution (20–60 min) showed three phases: mild inhibition → facilitation → slight facilitation. 

(2) Under 0 bar conditions, HDT reduced the mean bubble radii, though this effect exhibited marginal influence during the initial 10 min, while at 1 bar or 2 bar, HDT increased the mean bubble radii, with its impact gradually stabilizing with increasing HDT concentration within the first 10 min period.

(3) Ambient pressure governed effects of HDT on foam half-life: At 0 bar, half-life increased from 9 min 50 s to 11 min with HDT addition before stabilizing. At 1 bar or 2 bar, half-life decreased dose-dependently. Optimal formulation: AES 15%, HDT 4%, stabilizer 0.1%, and water 80.9%.

(4) For clays with PIs of 20–50, conventional foam agent maintains cohesion values at 4.7–19.5 kPa and adhesion amounts at 38–53 g, presenting significant clogging risks, whereas dispersed foam agent reduced cohesion to 1.8–4.3 kPa and clay adhesion amounts to 10–15 g, demonstrating that the dispersed foam agent significantly reduces clay cohesiveness, thereby inhibiting clogging formation. Furthermore, this study established optimal FIR for the dispersed foam agent corresponding to varying pressure conditions and PIs of clay.

In summary, this study developed a high-dispersibility foam agent by synergizing an anionic surfactant (AES) with a nonionic dispersant (HDT), achieving a breakthrough in mitigating clogging risks for EPB tunneling in highly cohesive clays. This study elucidates the pressure-dependent evolution of bubble size distribution and foam stability, demonstrating that HDT content critically modulates foam degradation dynamics within 0–2 bar pressure regimes. The cohesion of conditioned clays was reduced to 1.8–4.3 kPa, with adhesion amounts suppressed by >85%. Field validation in Jinan Metro Line 6 demonstrated a 133% increase in tunneling efficiency, while the proposed operational guidance framework (Figure 14) provides a science-based protocol for optimizing FIR under diverse geological and pressure conditions.

Future studies will focus on establishing a time-dependent rheological model that integrates foam half-life decay dynamics and bubble evolution mechanisms (Section 3.1 and Section 3.2) to predict critical clogging thresholds under varying advance rates. This pressure–time–rheology coupling framework will be validated through dynamic screw conveyor simulations and field monitoring.

## Figures and Tables

**Table 1:** The necessary properties of the above materials.

Materials	Physical State	Density (g/cm^3^)	Chemical Composition
AES	transparent viscous fluid	1.06	Sodium Alcohol Ether Sulfate
HDT	transparent liquid	1.20	Crosslinked Sodium Polyacrylate
foam stabilizer	transparent light-yellow viscous liquid	1.05	Cocamidopropyl Betaine

**Table 2:** Formulation of synthetic clay groups.

PI	Mass Ratio (Bentonite–Illite–Kaolin)	Moisture Content (%)
20	0.5:2:3	40
30	1:2:3	50
40	1.5:1:2	55
50	1:1:1	60

**Table 3:** The necessary properties of clay minerals.

Clay Mineral	Physical State	Density (g/cm^3^)	Chemical Composition	D_10_ (μm) ^1^	D_50_ (μm) ^1^	D_90_ (μm) ^1^
Bentonite	Light-yellow powder	2.20	Montmorillonite	1.708	5.297	23.650
Illite powder	Light-green powder	2.65	Illite	1.273	3.476	14.335
Kaolin	White powder	2.60	Kaolinite	0.895	2.260	9.037

^1^ Particle size analysis was conducted with reference to ISO 13320:2020.

**Table 4:** The PSDs of the four clay samples.

PI	D_10_ (μm) ^1^	D_50_ (μm) ^1^	D_90_ (μm) ^1^
20	0.951	2.582	10.970
30	0.982	2.896	13.630
40	1.054	3.157	13.910
50	1.117	3.550	15.360

^1^ Particle size analysis was conducted with reference to ISO 13320:2020.

**Table 5:** Field performance comparison with literature.

Study	Site/Stratum	Conditioning Materials	FIR	Key Field Results	Clogging Status
This study	Jinan Metro, silty clay	Dispersed foam	30%	Torque ↓42% (3000→1750 kN·m); advance rate ↑133%	Fully resolved
Wan et al. [23]	Changchun Metro, weathered mudstone	Foam + anti-clay agent	70%	Torque ↓~30% (3500→2500 kN·m); adhesion ↓88%	Partially resolved
Lu et al. [34]	Fuzhou Metro, coastal silty clay	Dispersed foam	40–50%	Torque ↓~15% (avg. 250 kN·m); foam usage ↓18.6%	Largely resolved
Jin et al. [35]	Fujian, gravel-clay	RSM-optimized foam	50%	Thrust ↓4.6%; torque ↓9%; cost ↓19%	Reduced clogging risk

## Data Availability

The original contributions presented in this study are included in the article. Further inquiries can be directed to the corresponding author.

## Appendix A

Appendix A describes the testing procedures for the foam evolution mechanism, the cohesion of conditioned clay, and the cutterhead rotation adhesion of conditioned clay.

### Appendix A.1

The testing process for the foam evolution mechanism is as follows [7,8]; the research program is shown in Table A1.

Step 1: Setup of foaming environment pressure.

If the test pressure is 0 bar, connect the right-side interface of the observation window directly to the atmospheric environment. Otherwise, connect the right side of the observation window to the foam degradation column. Use the foam degradation column to regulate the internal pressure of the observation window to the specified test pressure (1 bar or 2 bar).

Step 2: Foam generation.

Adjust the pressure control device to maintain the pressure gauge reading on the left side of the foam generator at the set value (e.g., 300 kPa). Regulate the gas flow meter and the foaming solution pump to maintain a constant FER of 15.

Step 3: Foam distribution image acquisition.

Once stable foam generation is achieved and the observation window is completely filled, simultaneously close both valves on the observation window. Immediately initiate image capture (as shown in Figure 2) of the foam within the observation window using the microscope. Continue image acquisition for a duration of 1 h.

### Appendix A.2

The testing process for the cohesion of conditioned clay is as follows [26]; the research program is shown in Table A1.

Step 1: Soil Sample Compaction

The prepared soil sample is thoroughly mixed with foam at a specified FIR. The mixture is then placed into the shear box using a layered compaction method. Each layer is compacted to a height of 1 cm. After placement, each layer is pre-compacted using a 1 kg weight to ensure uniform density. Upon completing pre-compaction for a layer, the next layer is added until the target height is achieved.

Step 2: Vertical Loading

I. Apply vertical pre-loading at 0.1 mm/min to ensure the vertical loading plate makes contact with the soil sample.

II. After contact is established:

a. Zero the vertical displacement gauge.

b. Apply the vertical load at the predetermined rate.

Step 3: Horizontal Shearing

I. Apply horizontal pre-loading at 0.1 mm/min to ensure the shear loading plate contacts the soil sample.

II. After contact is established:

a. Zero the horizontal displacement gauge.

b. Apply the shear load at the predetermined rate until 20 mm of shear displacement is reached. Terminate the test.

### Appendix A.3

The testing process for the cutterhead rotation adhesion of conditioned clay is as follows [27]; the research program is shown in Table A1.

Step 1: Soil Sample Compaction

The prepared soil sample is thoroughly mixed with foam at a specified foam injection ratio (FIR). The mixture is then placed into the soil container using a layered placement method, with each layer compacted to a height of 5 cm. After placement, each layer is pre-compacted using a 1 kg weight. Upon completing pre-compaction, the next layer is added until the total height reaches 20 cm.

Step 2: Tunnelling Module Installation

The soil-filled tunnelling module is installed in position. The miniature cutterhead is lowered to contact the improved soil surface. The internal air pressure of the tunnelling module is then set to the preset test pressure value.

Step 3: Clay Adhesion Test

The test is conducted under constant cutterhead rotation speed and advance rate:

Cutterhead rotation speed: 2 rpm

Advance rate: 40 mm/min

Excavation depth: 10 cm

After the completion of the excavation, the weight of clay adhered to the cutterhead is measured to evaluate the clay adhesion characteristics.

**Table A1:** The research program.

Test	Apparatus and Reference	Key Input Parameters	Output Metrics
foam evolution mechanism	foam testing system, Wu et al. [7]	(1) *p*: 0–2 bar (2) Time: 0–60 min (3) *c_d_*: 0%–10%	(1) proportion of small bubbles (2) mean radii of foam (3) foam half-life
cohesion of conditioned clay	unsaturated soil direct shear system, Peña Duarte [26]	(1) PI: 20–50 (2) foam or dispersed foam (3) FIR: 0–100%	(1) cohesion of conditioned clay
cutterhead rotation adhesion of conditioned clay	cutterhead rotation adhesion testing system, Jakobsen et al. [27]	(1) PI: 20–50 (2) foam or dispersed foam (3) FIR: 0–100% (4) *p*: 0–2 bar	(1) adhesion amount of conditioned clay (2) loss ratio of adhesion amount of conditioned clay

## Appendix B

Appendix B provides detailed data tables for the results of Section 3, Results and Discussion.

**Table A2:** Proportion of bubbles with r ≤ 0.05 mm.

*p* (bar)	*c_d_* (%)	Proportion of Bubbles with r ≤ 0.05 mm (%)						
		0 min	10 min	20 min	30 min	40 min	50 min	60 min
0	0	69.27	42.03	31.88	28.52	20.00	12.00	3.39
	2	76.38	40.32	31.02	25.98	19.00	13.00	6.68
	4	79.76	39.60	30.61	24.07	20.50	16.51	12.64
	6	80.77	40.68	32.74	30.46	23.13	16.01	9.33
	8	81.35	41.23	33.91	33.33	25.06	17.11	7.69
	10	81.51	42.21	34.00	33.62	27.00	17.12	5.09
1	0	84.05	65.07	60.36	58.33	53.98	51.08	47.77
	2	76.33	65.37	61.56	54.85	51.02	47.02	43.30
	4	72.66	65.93	62.77	51.32	44.93	38.11	32.28
	6	72.88	65.72	62.56	52.43	46.89	41.97	37.38
	8	73.44	65.58	62.41	53.59	49.95	46.01	43.27
	10	74.31	66.22	62.66	54.43	50.20	46.82	43.27
2	0	89.22	72.44	—	—	—	—	—
	2	76.31	72.89	—	—	—	—	—
	4	70.6	69.35	—	—	—	—	—
	6	70.67	69.12	—	—	—	—	—
	8	70.12	68.95	—	—	—	—	—
	10	69.95	68.78	—	—	—	—	—

**Table A3:** Mean radii of bubbles.

*p* (bar)	*c_d_* (%)	Mean Radii (mm)						
		0 min	10 min	20 min	30 min	40 min	50 min	60 min
0	0	0.07529	0.31635	0.56112	0.71089	0.85332	0.98213	1.12007
	2	0.07511	0.31041	—	—	—	—	—
	4	0.07507	0.30573	0.46072	0.62104	0.73053	0.82076	0.9201
	6	0.07657	0.30498	—	—	—	—	—
	8	0.07692	0.30395	—	—	—	—	—
	10	0.07698	0.30403	—	—	—	—	—
1	0	0.05171	0.12362	0.17131	0.22055	0.26011	0.31003	0.34032
	2	0.06049	0.15262	—	—	—	—	—
	4	0.06627	0.16365	0.22019	0.27048	0.33016	0.371316	0.40469
	6	0.06807	0.16363	—	—	—	—	—
	8	0.06796	0.16432	—	—	—	—	—
	10	0.06824	0.16461	—	—	—	—	—
2	0	0.04990	0.11509	0.16052	0.19133	0.21117	0.21801	0.23032
	2	0.05492	0.12366	—	—	—	—	—
	4	0.05747	0.12497	0.17039	0.22017	0.26035	0.29042	0.32007
	6	0.05766	0.12502	—	—	—	—	—
	8	0.05812	0.12483	—	—	—	—	—
	10	0.05834	0.12487	—	—	—	—	—

**Table A4:** Half-life of foam.

*p* (bar)	Half-Life (min)					
	0%	2%	4%	6%	8%	10%
0	9.88	10.57	10.97	10.98	10.97	10.88
1	15.15	15	13.02	12.8	12.75	12.67
2	16.47	15.97	14.62	14.03	13.83	13.53

**Table A5:** Cohesion of conditioned clay.

Conditioner		Foam				Dispersed Foam			
PI		20	30	40	50	20	30	40	50
Cohesion (kPa)	0%	15.00	23.00	31.29	38.59	15.00	23.00	31.29	38.59
	10%	14.50	22.20	30.18	36.11	10.87	17.10	23.25	29.70
	20%	10.90	19.20	28.25	33.62	1.80	10.31	17.37	22.20
	30%	6.80	15.40	24.76	30.78	1.20	2.10	12.09	15.40
	40%	4.70	10.21	20.35	26.00	1.10	1.40	3.83	9.90
	50%	4.60	10.19	16.88	19.60	1.10	1.38	2.84	4.35
	60%	4.60	10.19	16.01	19.50	1.10	1.35	2.61	3.72
	70%	4.60	10.15	15.79	19.48	1.10	1.35	2.34	3.71
	80%	4.60	10.15	15.8	19.47	1.10	1.35	2.30	3.70
	90%	4.60	10.15	15.82	19.46	1.10	1.35	2.28	3.70
	100%	4.50	10.15	15.73	19.46	1.10	1.35	2.29	3.70

**Table A6:** Adhesion amount of conditioned clay.

Conditioner		Foam			Dispersed Foam		
*p* (bar)		0	1	2	0	1	2
Adhesion amount (g)	0%	89	98	104	89	98	104
	10%	76	81	77	62	48	38
	20%	70	65	64	35	26	18
	30%	61	57	57	25	19	15
	40%	53	50	53	15	14	14
	50%	48	43	52	10	12	13
	60%	42	42	52	9	11	14
	70%	38	42	52	8	11	14
	80%	37	42	52	9	11	13
	90%	38	42	53	8	11	14
	100%	37	43	52	9	11	13

**Table A7:** Loss ratio of conditioned clay.

Conditioner		Foam			Dispersed Foam		
*p* (bar)		0	1	2	0	1	2
Loss ratio (%)	0%	0	0	0	0	0	0
	10%	14.61	17.35	25.96	30.34	51.02	63.46
	20%	21.35	33.67	38.46	60.67	73.47	82.69
	30%	31.46	41.84	45.19	71.91	80.61	85.58
	40%	40.45	48.98	49.04	83.15	85.71	86.54
	50%	46.07	56.12	50	88.76	87.76	87.5
	60%	52.81	57.14	50	89.89	88.78	86.54
	70%	57.30	57.14	50	91.01	88.78	86.54
	80%	58.43	57.14	50	89.89	88.78	87.5
	90%	57.30	57.14	49.04	91.01	88.78	86.54
	100%	58.43	56.12	50	89.89	88.78	87.50
